# Supplementary material for: Plasma-derived extracellular vesicles from Plasmodium vivax patients signal spleen fibroblasts via NF-kB facilitating parasite cytoadherence
Source: Nat Commun. 2020 Jun 2;11:2761. doi: 10.1038/s41467-020-16337-y (PMC7265481; doi:10.1038/s41467-020-16337-y)
Supplement: Supplementary file 1 — Supplementary Information [file 41467_2020_16337_MOESM1_ESM.pdf]

Supplementary Information

Plasma-derived extracellular vesicles from *Plasmodium vivax* patients signal spleen fibroblasts via NF-κB facilitating parasite cytoadherence

Haruka et al.

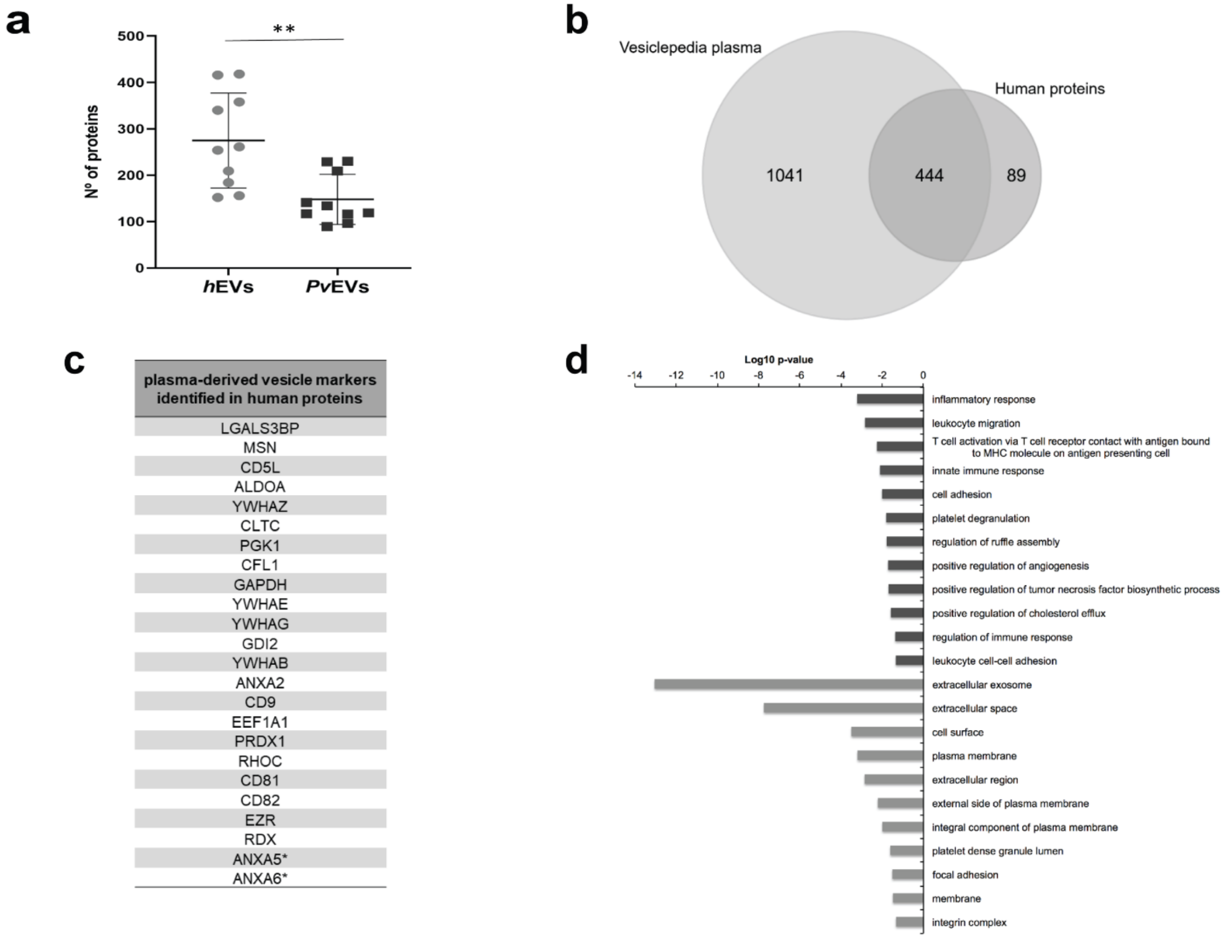

Supplementary Figure 1. Proteomic characterization of the human component of *Pv*EVs and *h*EVs. (a) Comparison of total number of human proteins identified in healthy donors and *P. vivax* patients (*n* =10, biologically independent samples). Data show mean ± SD. Two-sided, Mann-Whitney test, ***p* =0.0023 (GraphPad). Source data are provided as a Source Data file. **(b)** Venn diagram comparison of the 533 human proteins identified in *Pv*EV*s* and *h*EV*s* with the plasma proteins compiled in the public repository of EV cargo Vesiclepedia [40]. 444 proteins were detected in the intersection. **(c)** Plasma-derived EVs markers detected among the human proteins identified in *Pv*EVs and *h*EVs. Two proteins exclusively present in healthy donors are highlighted (*). **(d)** Gene ontology (GO)-enrichment analysis of the 33 proteins exclusively associated to *Pv*EVs. Bar plot shows the more significant GO terms on the category Biological process (black bars) and Cellular component (gray bars). Source data are provided as a Source Data file and Supplementary Data 2, 3, and 4.

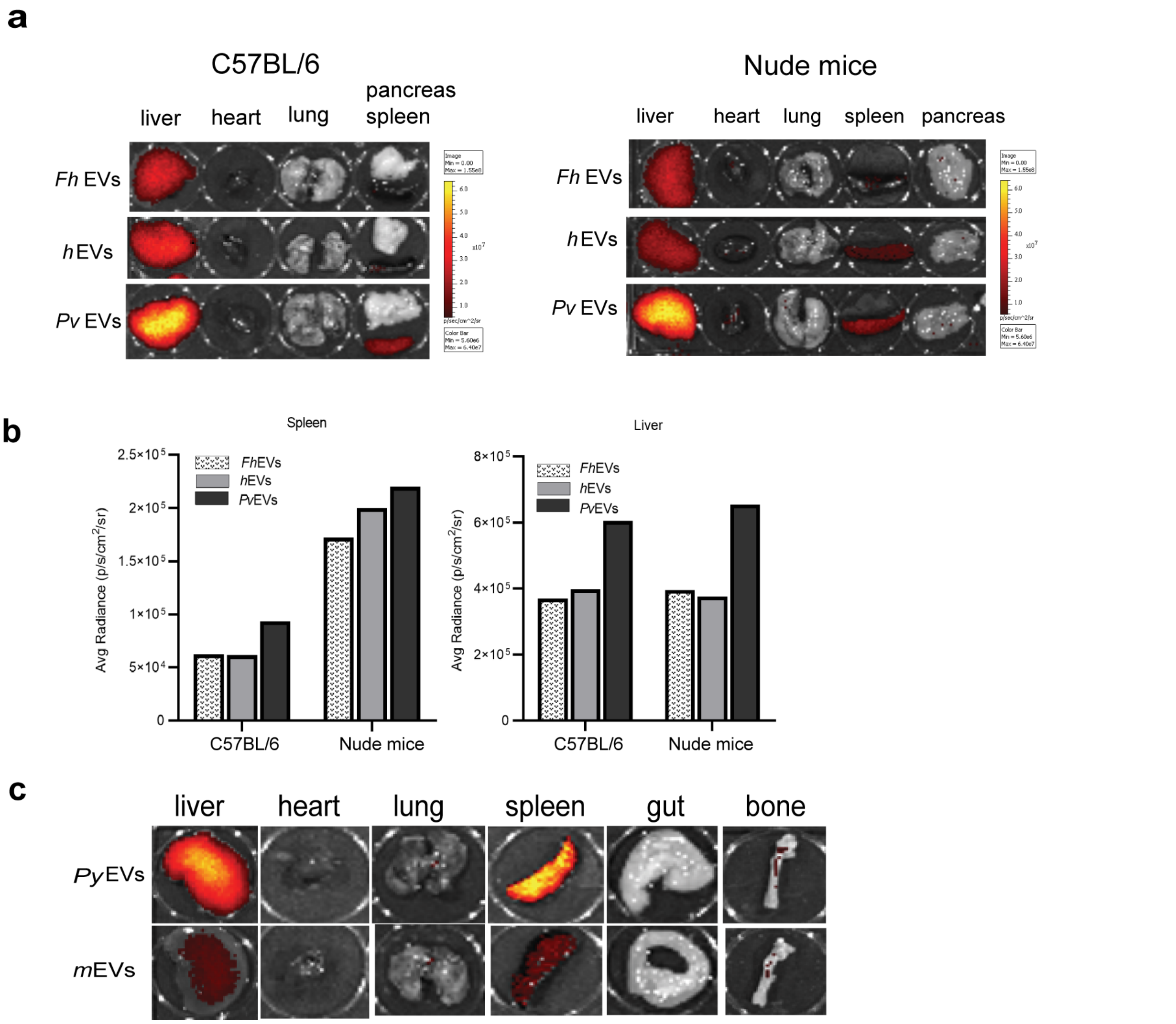

Supplementary Figure 2. *In vivo* distribution of EVs in mice injected with labelled EVs. (a) Representative IVIS-Spectrum images of the *in vivo* distribution of pooled EVs from *P. vivax* patients (*Pv*EVs), healthy human volunteers (*h*EVs), and *Fasciola hepatica* (*Fh*EVs), after injection into C57BL/6 mice (left) and in nude mice (right) (n = 1). After 1h, mice were sacrificed to analyze different organs as indicated. **(b)** Quantification of EVs in liver and spleen. Source data are provided as a Source Data file. **(c)** Reticulocyte-derived EVs from mice infected with *P. yoelii*-infected (*Py*EVs) and plasma-derived EVs from healthy mice (*m*EVs) were injected via the retro-orbital venous sinus into C57BL/6 mice (n = 3). After 1 h, mice were sacrificed and organs were harvested for the analysis. The images of each organ were captured by IVIS-SPECTRUM imaging system (PerkinElmer).

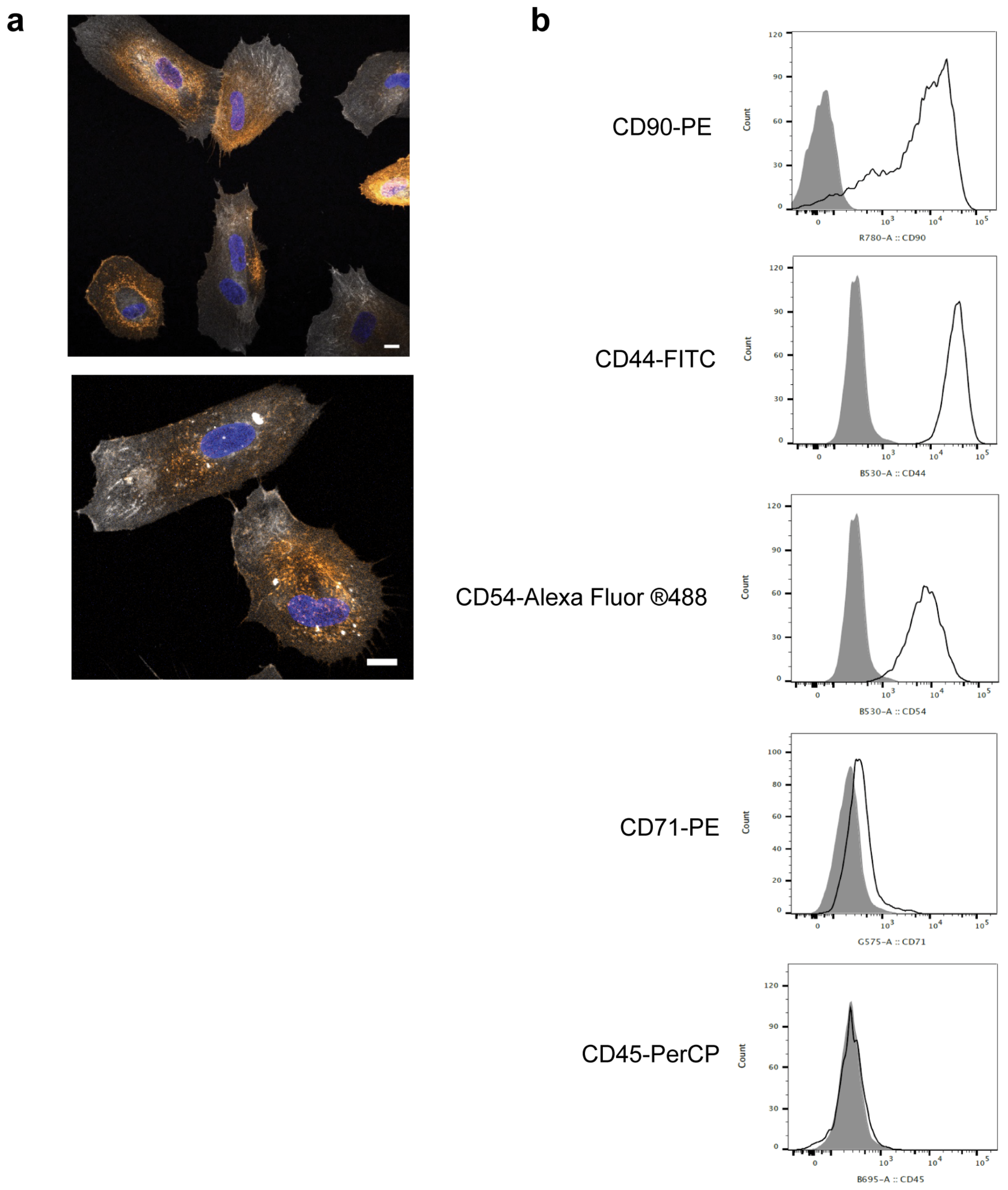

Supplementary Figure 3. Characterization of human spleen fibroblasts (*h*SFs). (a) Confocal images of *h*SFs. Nuclei were stained with Hoechst® 33342 (blue), cell membranes with Cell Mask™ (white) and ICAM-1 with Alexa 488-conjugated ICAM-1 (orange) with a 40x/NA1.30 (upper) and 63x/NA1.40 (lower) oil-immersion objectives. Scale bar: 10µm. Image representative of three independent experiments. **(b) Expression of the indicated molecules in *h*SFs measured by LSR-Fortessa cytometer.** The background staining (gray histograms) are shown in each histogram.

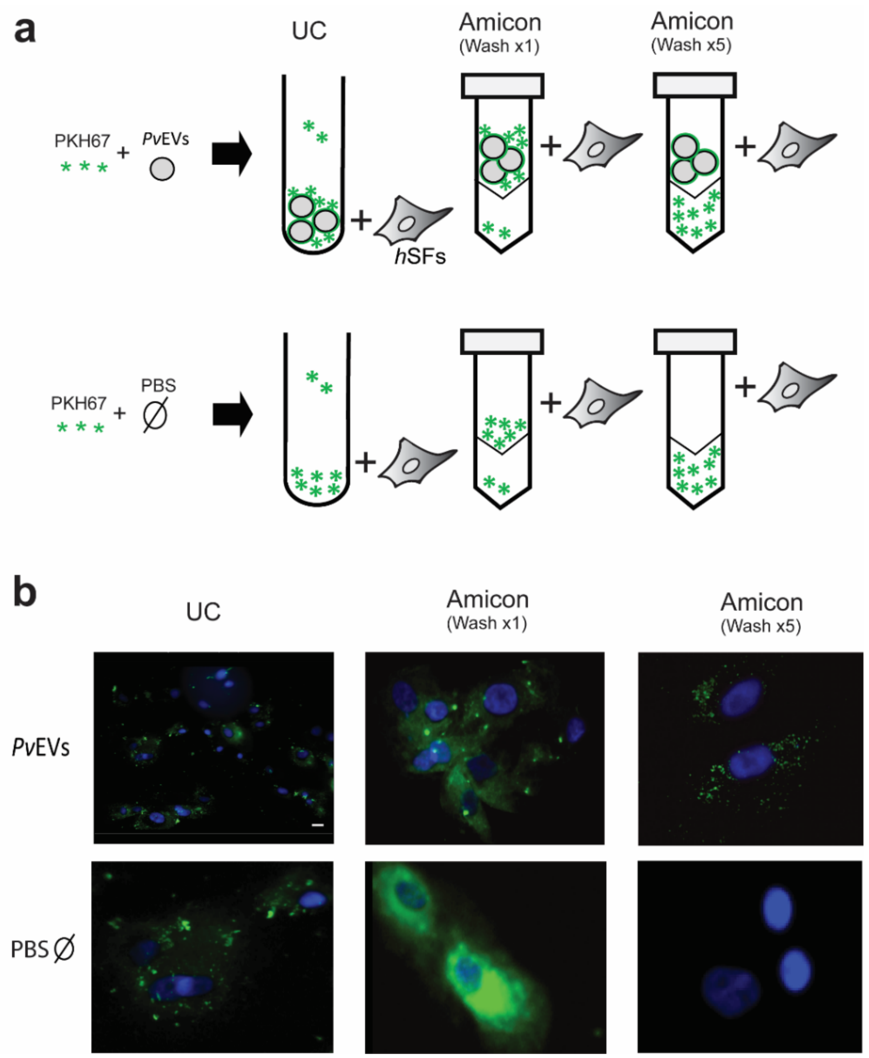

Supplementary Figure 4. EVs-staining and washing methods for confocal microscopy analysis. (a) Removal of excess dye. Pooled *Pv*EVs or equal amount of PBS were stained with PKH67 (green). In order to remove the excess dye, stained EVs and stained PBS were washed by three different methods: 2 times washed by ultracentrifugation (UC), 1 time washed by Amicon 100kD cutoff filters, and 5 times washed by Amicon 100-kDa cutoff filters. EVs and PBS were incubated with *h*SFs at 37°C for 2 h. Small green starts show unbounded PKH67 dyes, and gray circle encircled by green line show PKH67-stained EVs. **(b) Confocal images of the different washing methods.** *h*SFs incubated with PKH67-stained EVs or PBS washed by three different methods. Nucleus were stained with Hoechst® 33342 (blue). Scale bar: 10µm. Image representative of three independent experiments.

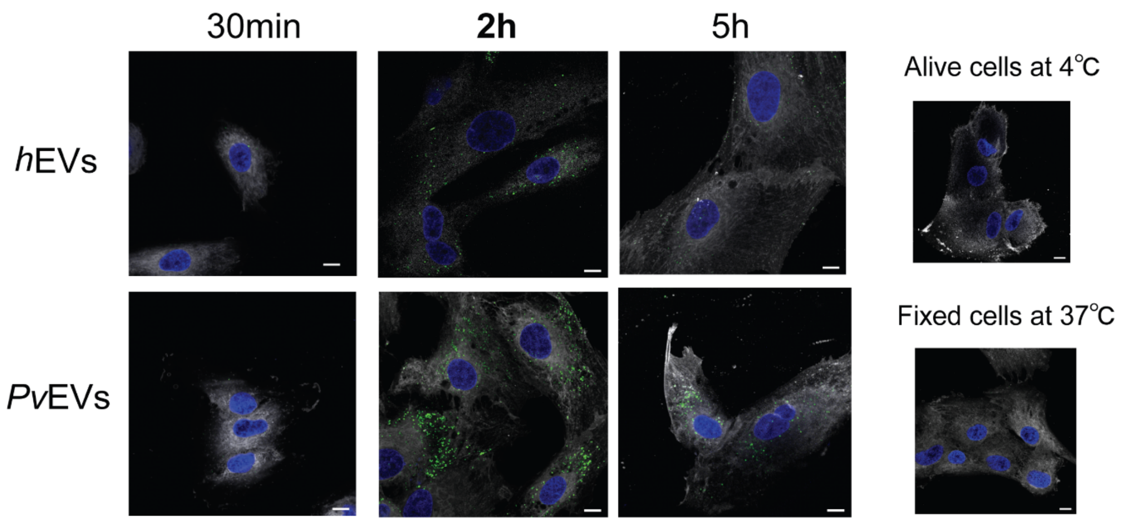

Supplementary Figure 5. Uptake of EVs by *h*SFs. Left panel. Confocal images of the uptake time course of PKH67-stained pooled *Pv*EVs (green) and *h*EVs (green) individually incubated with *h*SFs at 37°C. **Right panel.** Confocal images of controls, PKH67-stained pooled *Pv*EVs incubated with *h*SFs at 4°C or with fixed *h*SFs at 37°C for 2 h. Nucleus were stained with Hoechst® 33342 (blue) and cell membrane were stained with Cell Mask™ (white). Scale bar: 10µm. Image representative of three independent experiments.

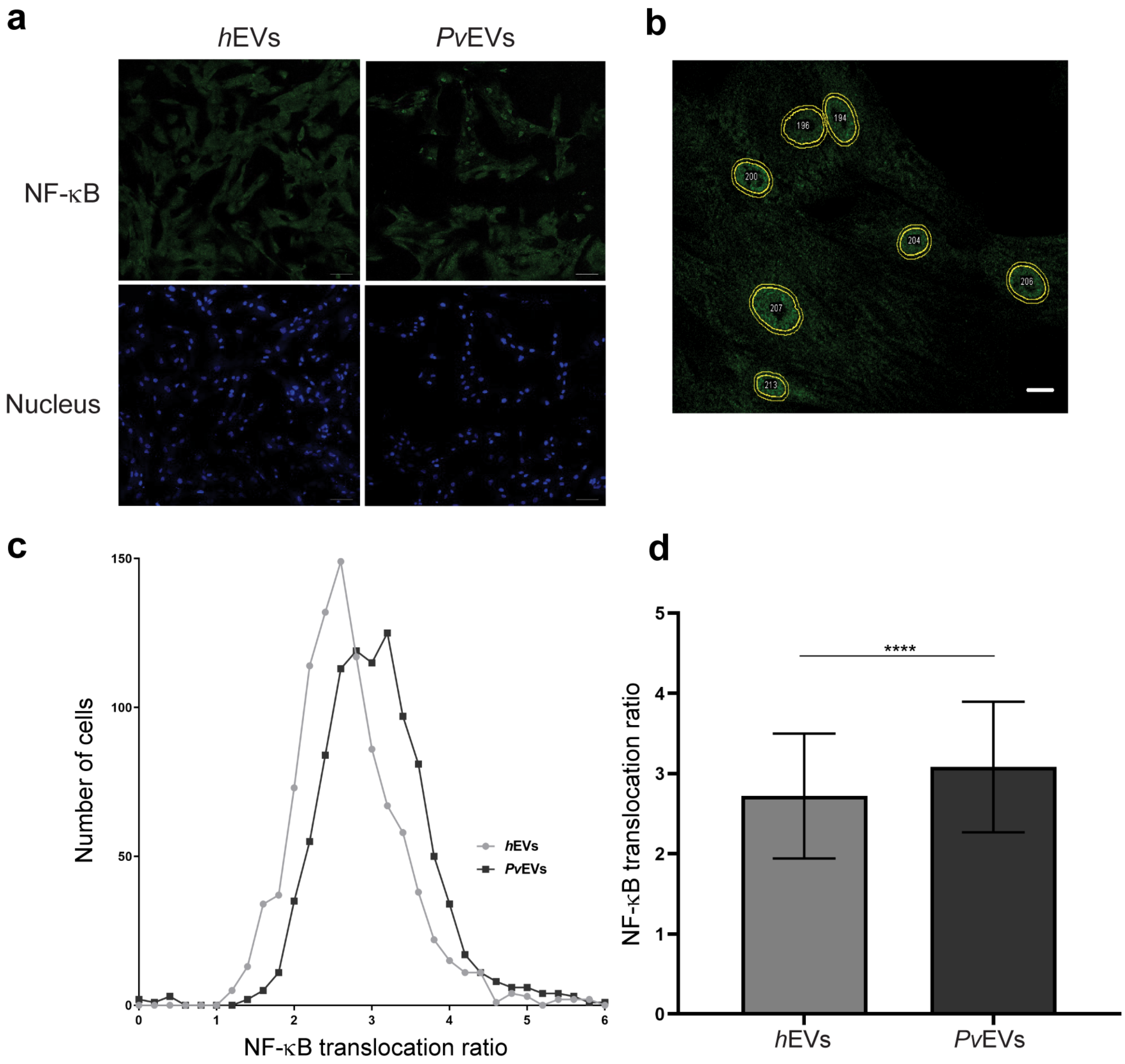

Supplementary Figure 6. Quantification of NF-κB translocation (a) Confocal images of *h*SFs. *h*SFs were incubated with pooled *Pv*EVs or *h*EVs for 30 min. Nuclear translocation was determined by nucleus staining (blue) and NF-κB staining (green) (x40oil, 5x5 tiled image). Scale bar: 100 μm. Image representative of three independent experiments. **(b) Nuclear and perinuclear regions**. Image example to define nuclear and perinuclear regions of individual cells (yellow) determined by Macro. Scale bar: 10 μm. Image representative of three independent experiments. **(c)** NF-κB translocation ratio ($intD_{Nuclear}/IntD_{Perinuclear}$) for each cell (*n* = 1000) indicating normal distribution. **(d) Quantification of the NF-κB translocation ratio**. Data show mean ± SD (*n* = 1000). Unpaired and two-sided, *t*-test, *****p* < 0.0001 (GraphPad). Source data are provided as a Source Data file.

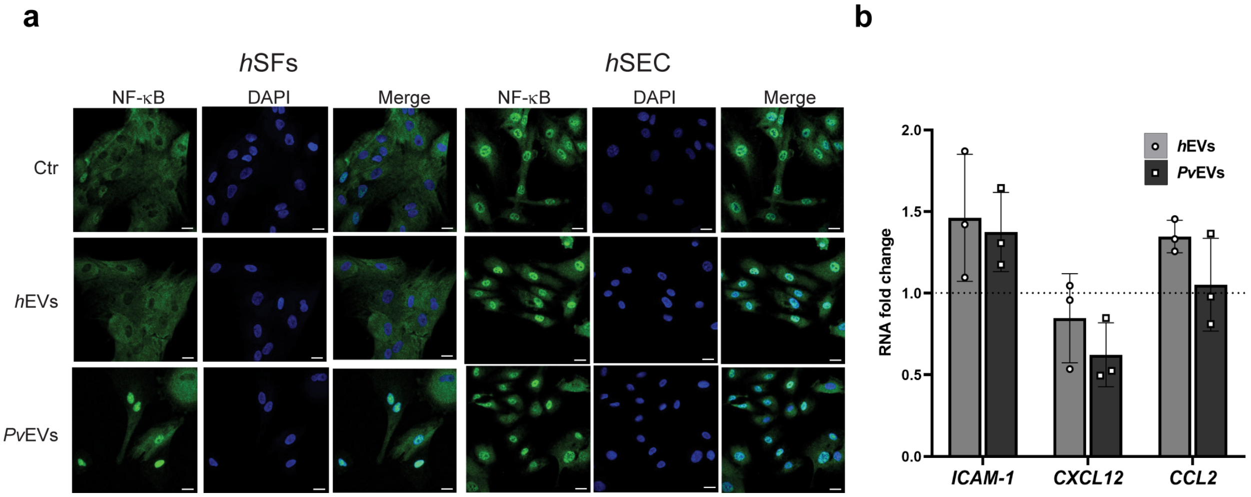

Supplementary Figure 7. NF-κB nuclear translocation and *ICAM-1* expression in human spleen endothelial cells (*h*SEC). (a) Nuclear translocation*.* Human spleen fibroblasts (*h*SFs) or human spleen endothelial cells (*h*SEC) were incubated with media alone (Ctr) or with pooled *Pv*EVs or *h*EVs for 30 min. Nuclear translocation was determined by nucleus staining (blue) and NF-κB staining (green). Scale bar: 20 μm. Image representative of two independent experiments. **(b) ICAM-1 expression.** Quantitative RT-PCR was performed to analyze the expression of *ICAM-1*, *CXCL12*, and *CCL2* in *h*SEC. *GNB2L1* was used as endogenous control. All data show means of the fold-change relative to control values ± SD (technical replicates, *n* = 3). Unpaired and two-sided, t-test. No significant value was observed in any genes (GraphPad). Source data are provided as a Source Data file.

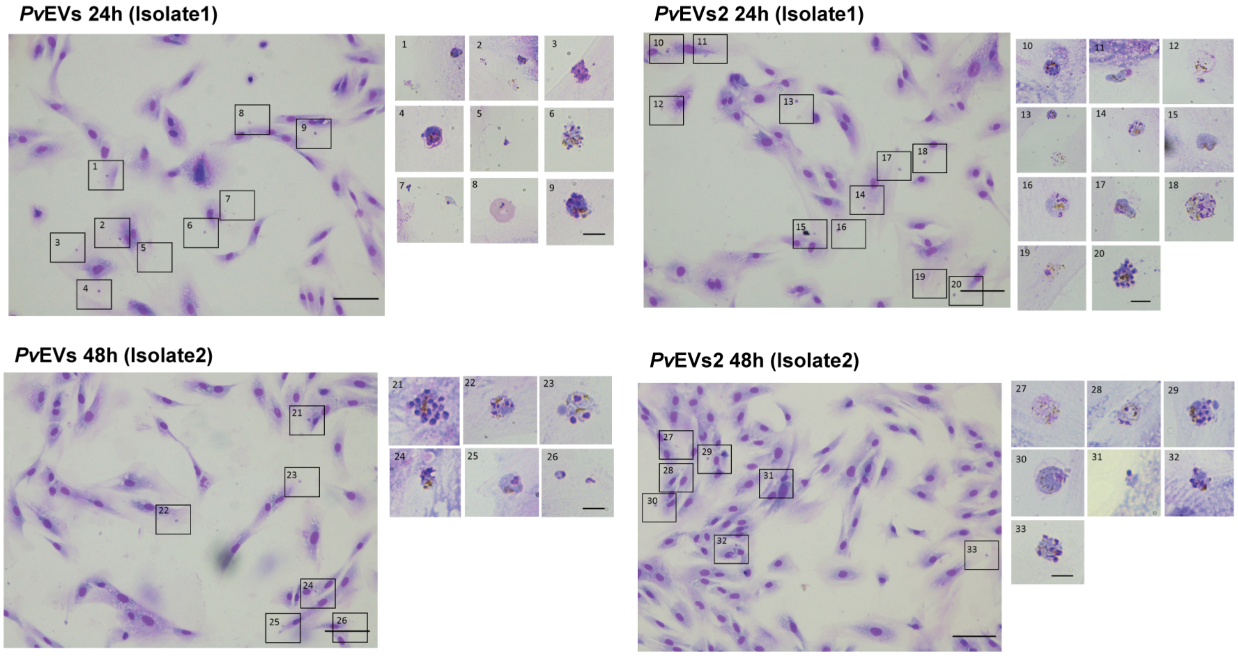

Supplementary Figure 8. *P. vivax*-infected reticulocytes binding to *h*SFs. *h*SFs were pre-treated with pooled *Pv*EVs or *Pv*EVs2 for 24 h or 48 h and incubated with two different isolates (1 or 2) for 45 min (*n* = 4 - *Pv*EVs). Phase contrast images of Giemsa-staining *h*SFs were captured by optical microscopy. Left: x20/0.40. Scale bar: 50 µm. Right (1-33): x100/1.25 oil. Scale bar: 5 µm. Images (right) are the zoom of each square in images (left). Each image represents each independent experiment as indicated.

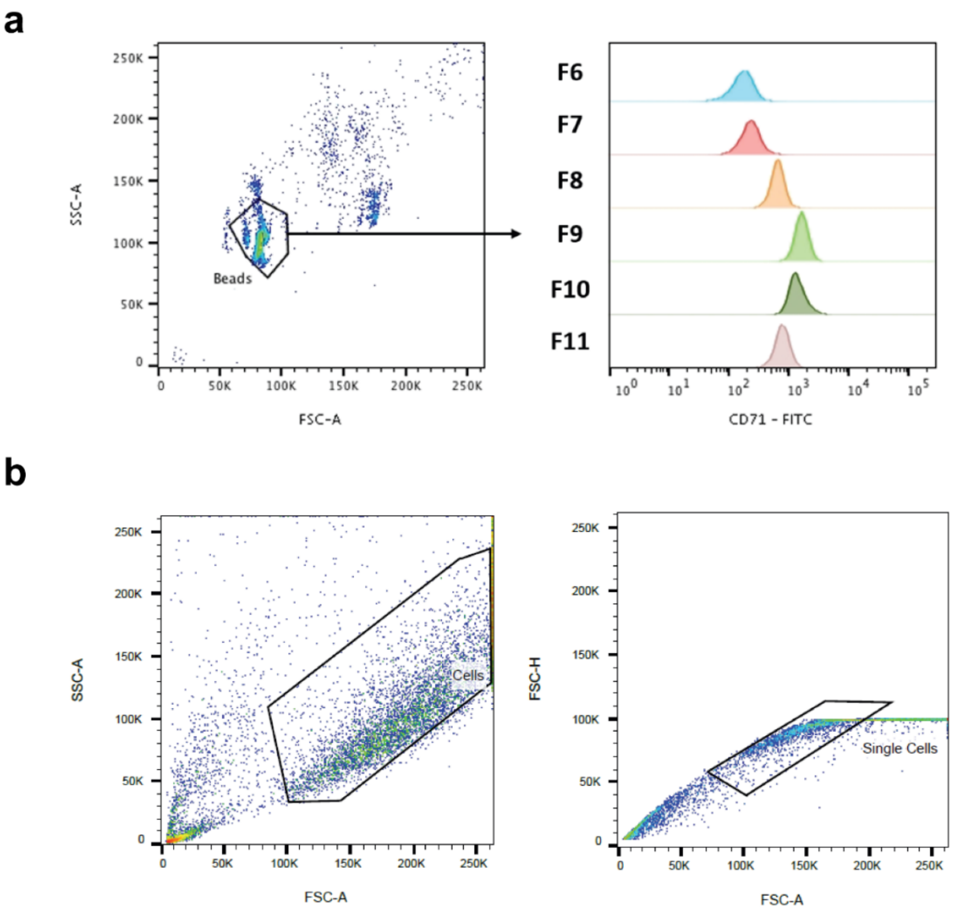

Supplementary Figure 9. Gating strategy of FACS analysis. Flow cytometry data showed in pseudocolor plots, with blue and green corresponding to areas of lower particle density, yellow representing mid-range density, and red and orange are areas of high particle density. K, thousands. In total, 10.000 events/sample were analyzed. **(a) Gating strategy for bead-based flow cytometry analysis.** EV-coupled beads were first gated according to FSC-A/SSC-A. Expression of markers (e.g. CD71) was compared among different fractions using the median of FITC of the beads population **(b) Gating strategy for FACS analysis.** The population of cells was selected by FSC-A/SSC-A and cell debris and dead cells were excluded. Afterwards, singlets were selected by FSC-A/FSC-H. For *h*SFs characterization, compare the positivity of interest markers with unstained cells using histogram (corresponding fluorescence/count). For ICAM-1 expression, median of B530-A subset (correspond to Alexa Fluor 488) was used for the quantification.

Gene Name	Assay ID
GNB2L1	Hs00272002_m1
ACTA2	Hs00426835_g1
GM-CSF	Hs00929873_m1
VEGFa	Hs00900055_m1
CXCL12	Hs00171022_m1
FBG8	Hs00171832_m1
ICAM-1	Hs00164932_m1
IL-6	Hs00985639_m1
IL-10	Hs00961622_m1
CCL2	Hs00234140_m1
TLR4	Hs00152939_m1
TRL7	Hs00152971_m1
TRL9	HS00152973_m1

Supplementary Table 1. List of the gene names and corresponding Assay ID used for qRT-PCR analysis. Assay ID is according to TaqMan® Gene Expression Assays (Thermo Fisher Scientific). Sequences of the primers are not available from the company.
