# Supplementary material for: Plasma-derived extracellular vesicles from Plasmodium vivax patients signal spleen fibroblasts via NF-kB facilitating parasite cytoadherence
Source: Nat Commun. 2020 Jun 2;11:2761. doi: 10.1038/s41467-020-16337-y (PMC7265481; doi:10.1038/s41467-020-16337-y)
Supplement: Supplementary file 3 — Reporting Summary [file 41467_2020_16337_MOESM3_ESM.pdf]

nature research

Corresponding author(s): DBPR NCOMMS-19-20723B

Last updated by author(s): Apr 10, 2020

Reporting Summary

Nature Research wishes to improve the reproducibility of the work that we publish. This form provides structure for consistency and transparency in reporting. For further information on Nature Research policies, see Authors & Referees and the Editorial Policy Checklist .

Statistics

For all statistical analyses, confirm that the following items are present in the figure legend, table legend, main text, or Methods section.

n/a	Confirmed	
☐	☒	The exact sample size (n) for each experimental group/condition, given as a discrete number and unit of measurement
☐	☒	A statement on whether measurements were taken from distinct samples or whether the same sample was measured repeatedly
☐	☒	The statistical test(s) used AND whether they are one- or two-sided<br>*Only common tests should be described solely by name; describe more complex techniques in the Methods section.*
☒	☐	A description of all covariates tested
☐	☒	A description of any assumptions or corrections, such as tests of normality and adjustment for multiple comparisons
☐	☒	A full description of the statistical parameters including central tendency (e.g. means) or other basic estimates (e.g. regression coefficient) AND variation (e.g. standard deviation) or associated estimates of uncertainty (e.g. confidence intervals)
☒	☐	For null hypothesis testing, the test statistic (e.g. F, t, r) with confidence intervals, effect sizes, degrees of freedom and P value noted<br>*Give P values as exact values whenever suitable.*
☒	☐	For Bayesian analysis, information on the choice of priors and Markov chain Monte Carlo settings
☒	☐	For hierarchical and complex designs, identification of the appropriate level for tests and full reporting of outcomes
☒	☐	Estimates of effect sizes (e.g. Cohen's d, Pearson's r), indicating how they were calculated

Our web collection on statistics for biologists contains articles on many of the points above.

Software and code

Policy information about availability of computer code

Data collection

Nanoparticle tracking NanoSight LM10 (Malvern Instruments Ltd) was used to determine the number and size distribution of particles
LI-COR Odyssey Infrared Imaging System was used to detect signal of western blots and Burgundy-labelled-EVs.
IVIS-SPECTRUM imaging system (PerkinElmer) was used for in vivo distribution
Confocal images were acquired on a Zeiss LSM 710 Confocal Module coupled to the Zeiss Axio Observer Z1 microscope
STED images were acquired on Leica SP8 STED 3X microscope
mRNA quantification were analyzed using an Agilent 2100 Bioanalyze
Quantitative real-time PCR reactions were performed with RT-qPCR Light Cyler 480 Roche system (Life science).
Images of binding assay were captured using optical microscopy (Eclipse Ci-L, Nikon)

Data analysis

For peptide and protein identification on the mass spectrometry files we used Mascot v2.5.1 (http://www.matrixscience.com) on Proteome DiscovererTM Software V2.0 (Thermo Fischer Scientific).

FunRich v3.1.3 was used to perform comparisons among the MS-identified data and to compare to the public repository of EV cargo Vesiclepedia.

Gene ontology (GO) enrichment analysis of the MS-identified data was performed with the Database for Annotation, Visualization and Integrated Discovery (David 6.8)

NTA software (version 3.2) was used for NTA measurement to quantify EVs
FacsVerse cytometer (BD) was used for beads-based FACS analysis to characterize EVs.

LSR-Fortessa cytometer (BD) was used for FACS analysis to characterize hSFs and its ICAM-1 expression.

FlowJo software v10 for the analysis of FSC files derived from flow cytometry experiments performed over EV-coupled beads and hSFs.

Quantification Software of images derived from IVIS-SPECTRUM imaging system (PerkinElmer) was used to quantify the signals of organs in biodistribution
Image J –NIH (version 1.52j) software was used for western blot and EVs quantification of confocal microscopy images
ZEN black 2012 (ZESSI microscopy) was used in image editing for confocal microscopy.

FiJi distribution of ImageJ (https://fiji.sc/) was used in image editing for STED microscopy and NF-kB quantification assay.

Quantitative real-time PCR reactions were analyzed by RT-qPCR Light Cyler 480 Roche system (Life science).

Statistics was performed using GraphPad Prism software (Version 8.3.0).

For manuscripts utilizing custom algorithms or software that are central to the research but not yet described in published literature, software must be made available to editors/reviewers. We strongly encourage code deposition in a community repository (e.g. GitHub). See the Nature Research guidelines for submitting code & software for further information.

Data

Policy information about availability of data

All manuscripts must include a data availability statement. This statement should provide the following information, where applicable:

- Accession codes, unique identifiers, or web links for publicly available datasets
- A list of figures that have associated raw data
- A description of any restrictions on data availability

The mass spectrometry proteomics data have been deposited to the ProteomeXchange Consortium via the PRIDE partner repository with the dataset identifier PXD018337.

The source data underlying Figs. 1a, c ,e, 2a–c, 3a-b, d, 4a and Supplementary Figs 1a, 2b and 7b are provided as a Source Data file. All the other data supporting the findings of this study are available in the main text or in supplementary materials.

Code: The ImageJ macro used for NF-kB translocation evaluation was deposited in Zenodo open-access repository with code 3555213 [https://doi.org/10.5281/zenodo.3555213] under Creative Commons Attribution 4.0 International license.

"Raw mass spectrometry data files were analyzed with the database search algorithm Mascot v2.5.1 (http://www.matrixscience.com) on the Proteome Discoverer software v2.0 (Thermo Fischer Scientific) using a customized protein P. vivax database including all sequences from the strains deposited at UniProt and the UniProt human database, downloaded on November 2016 (https://www.uniprot.org).

The filtered list of human proteins were compared to the public extracellular vesicle Vesiclepedia plasma protein database 42 (http://www.microvesicles.org/) and intersections were represented using the FunRich tool 43."

Field-specific reporting

Please select the one below that is the best fit for your research. If you are not sure, read the appropriate sections before making your selection.

☒ Life sciences ☐ Behavioural & social sciences ☐ Ecological, evolutionary & environmental sciences

For a reference copy of the document with all sections, see nature.com/documents/nr-reporting-summary-flat.pdf

Life sciences study design

All studies must disclose on these points even when the disclosure is negative.

Sample size	"No sample size-calculation was performed prior to experiments, as no preliminary data was available on effect size and variation."
Data exclusions	No data were excluded
Replication	Technical replicates of all experiments were performed, excepting for the in vivo distribution in mice and binding of parasites to human spleen fibroblast where biological replicates instead of technical replicates were performed due to, respectively, R3 principle and scarcity of human samples from infected patients with high parasitemias<br>Fig. 1a, b, Supplementary Fig.1 analyzed ten biologically independent samples.<br>Fig. 2a, Supplementary Fig. 2 analyzed four biologically independent animals.<br>Fig. 1c, 2b-d, 3c, Supplementary Fig.3, 4, 5, 6 were performed in three independent experiments.<br>Supplementary Fig.7 were performed in two independent experiments.<br>Fig. 1d,e Fig. 3a,b,d were performed in three technical replicates<br>Fig. 4a, Supplementary Fig.8 were performed in four independent experiment

All attempts at replication were successful.

Randomization

Samples for proteomics analysis were randomized. For the rest of experiments randomization was not performed as experiments used pools of samples from Brazil and from Colombia. Moreover, for binding experiments only parasites capable of maturation in vitro and obtained from infected patients were used.

Blinding

In all these studies, several independent experiments or technical triplicates were performed and measurements taken at the same time using the same machine settings.

Reporting for specific materials, systems and methods

We require information from authors about some types of materials, experimental systems and methods used in many studies. Here, indicate whether each material, system or method listed is relevant to your study. If you are not sure if a list item applies to your research, read the appropriate section before selecting a response.

Materials & experimental systems

n/a	Involved in the study
☐	☒ Antibodies
☐	☒ Eukaryotic cell lines
☒	☐ Palaeontology
☐	☒ Animals and other organisms
☐	☒ Human research participants
☒	☐ Clinical data

Methods

n/a	Involved in the study
☒	☐ ChIP-seq
☐	☒ Flow cytometry
☒	☐ MRI-based neuroimaging

Antibodies

Antibodies used

1. Anti-CD9 antibody [VJ1/20] - Hybridoma supernatants
2. Anti-CD63 antibody [TEA3/10] - Hybridoma supernatants
3. Anti-CD81 antibody [5A6] - Hybridoma supernatants
4. Anti-GAL3 antibody – Abcam Cat#ab84036
5. Anti-CD5L antibody – Abcam Cat#ab45408
6. Anti-CD71 antibody – Abcam Cat#ab84036
7. Goat anti-Rabbit IgG (H+L) Cross-Adsorbed Secondary Antibody, Alexa Fluor 488 – Thermo Fisher Scientific Cat#A11008
8. Goat F(ab')2 Anti-Mouse IgG(H+L), Human ads-FITC - Southern Biotech Cat#1032-02
9. Rabbit anti-PvPHIST81 serum: (Akinyi S, et al. 2012)
10. Rabbit anti-PvMSP3 27 serum: (Jaiang J, et al. 2013)
11. IRDye® 680LT Goat anti-Rabbit IgG Secondary Antibody - LI-COR Biosciences Cat#925-68021,
12. Anti-CD90-PE/Cy7 antibody [5E10] – Biolegend Cat#328124
13. Anti-CD44-FITC antibody [KM201] – Abcam Cat#ab25340
14. Anti-CD54-AlexaFlour488 antibody [HCD54] - Biolegend Cat#322714
15. Anti-CD71-PE antibody [AC102] - Miltenyi Biotec Cat#130-091-728
16. Anti-CD45-PerCP antibody [2D1] - BD biosciences Cat#345809
17. Anti-NF-kB p65 Recombinant Polyclonal Antibody [4-2HCLC] –Thermo Fisher Scientific Cat#710048
18. Goat anti-Rabbit IgG (H+L), Superclonal™ Recombinant Secondary Antibody, Alexa Fluor 488 – Thermo Fisher Scientific Cat#A27034

Validation

All of the commercially available antibodies were manufactured and validated in accordance with their specifications.
4: https://www.abcam.com/transferrin-receptor-antibody-ab84036.html
5: https://www.abcam.com/cd5lct-2-antibody-ab45408.html
6: https://www.abcam.com/transferrin-receptor-antibody-ab84036.html
7: https://www.thermofisher.com/antibody/product/Goat-anti-Rabbit-IgG-H-L-Cross-Adsorbed-Secondary-Antibody-Polyclonal/A-11008
8: https://www.southernbiotech.com/?catno=1032-02&type=Polyclonal#&panel1-2
11: https://www.licor.com/bio/reagents/irdye-680lt-goat-anti-rabbit-igg-secondary-antibody
12: https://www.biocompare.com/9776-Antibodies/3722983-PE-Cy7-anti-human-CD90-Thy1/
13: https://www.abcam.com/cd44-antibody-km201-ab25340.html
14: https://www.biolegend.com/en-us/products/alexa-fluor-488-anti-human-cd54-antibody-3550

15: https://www.miltenyibiotec.com/US-en/products/macs-flow-cytometry/antibodies/primary-antibodies/cd71-antibodies-human-ac102-1-11.html#gref
16: https://www.bdbiosciences.com/eu/reagents/clinical/reagents/single-antibodies/cd45-percp-2d1/p/345809
17: https://www.thermofisher.com/antibody/product/NFkB-p65-Antibody-clone-4-2HCLC-Recombinant-Polyclonal/710048
18: https://www.thermofisher.com/antibody/product/Goat-anti-Rabbit-IgG-H-L Secondary-Antibody-Recombinant-Polyclonal/A27034

1: Anti-CD9 antibody, 2:Anti-CD63 antibody, and 3:Anti-CD81 antibody were validated in-house.
9: Rabbit anti-PvPHIST81 serum and 10: Rabbit anti-PvMSP3 27 serum were validated in previous publications (Akinyi S, et al., Mol Microbiol 84, 816-831 (2012) and (Jiang J, et al., PLoS One 8, e63888 (2013).

Eukaryotic cell lines

Policy information about cell lines

Cell line source(s)	Human spleen fibroblasts (hSFs) were obtained from long term splenocytes culture from deceased transplantion donors. Human spleen endothelial cells (hSEC) were purchased from ScienCell (Cat. #5500).
Authentication	Phenotype of hSFs were characterized by flow cytometer using CD90-PE/Cy7 (5E10, Biolegend) 1/100; CD44-FITC (KM201,Abcam) 1/100; CD54-AlexaFlour488 (HCD54, Biolegend) 1/400; CD71-PE (AC102, Miltenyi Biotec) 1/400; and CD45-PerCP (2D1, BD biosciences) 1/50, and by confocal microscopy using Hoechst® 33342 (Thermo Fisher), Cell Mask™ (Thermo Fisher), and CD54-AlexaFlour488 (HCD54, Biolegend) 1/400. hSEC were characterized by immunofluorescence with antibodies specific to VWF/Factor VIII and CD31 (PECAM) in Scien Cell.
Mycoplasma contamination	Mycoplasma contamination was checked and tested negative in all cell line experiments
Commonly misidentified lines (See ICLAC register)	No commonly misidentified cell lines were used in the study.

Animals and other organisms

Policy information about studies involving animals; ARRIVE guidelines recommended for reporting animal research

Laboratory animals	Six weeks-old C57BL/6 males and a six weeks-old male nude mouse were used. Mice were housed in cages with enrichment items located in ventilated racks at 21±1 °C and 50–60% humidity, with 12 h-light/12-h dark cycles. Food and water were provided ad libitum.
Wild animals	This study did not involve wild animals
Field-collected samples	Parasite samples were obtained from field isolates of individual P. vivax patients during their acute attack. Samples were obtained and processed at the BL2 laboratories of Fundaçao de Medicna Tropica Dr. heitor Vieira Dourado, Amazon, Brazil, Grupo de Investigaciones Microbiológicas y Biomédicas de Córdoba-GIMBIC, Universidad de Córdoba, Monteria, Colombia, and Mahidol Vivax Research Unit, Faculty of Tropical Medicine, Mahidol University, Bangkok, Thailand. Due to the scarcity of these samples, they were completely used at the end-of-experimental protocols.
Ethics oversight	Experiments were approved by the ISCIII Ethical Committee and Comunidad Autonoma de Madrid.

Note that full information on the approval of the study protocol must also be provided in the manuscript.

Human research participants

Policy information about studies involving human research participants

Population characteristics	Adult patients presenting positive diagnosis for vivax malaria (microscopy) were enrolled at endemic regions of Brazil (Manaus), Colombia (Tierralta) and Thailand (Mae Sod), after signed informed consent. All participants were treated according to recommendations for treatment of uncomplicated P. vivax malaria.
Recruitment	The only recruitment criterium was that participants were adults presenting positive diagnosis for vivax malaria (microscopy). Therefore, no selection-bias can impact the results.

Ethics oversight

Plasma from P. vivax patients used in this study were collected at the tertiary Hospital of the Fundaçao de Medicina Tropical Dr. Heitor Vieira Dourado (FMT-HVD), in the Amazon State, Brazil and in the E.S.E Hospital San José de Tierralta, Colombia. These studies were approved, respectively, by the local ethical committees of FMT-HVD in Brazil and Universidad de Córdoba, Monteria in Colombia and written informed consent was obtained from all the participants. Samples from healthy donors were collected at the Hospital Germans Trias i Pujol, Badalona, Spain, after expressed consent from the donors.

Note that full information on the approval of the study protocol must also be provided in the manuscript.

Flow Cytometry

Plots

Confirm that:

- [x] The axis labels state the marker and fluorochrome used (e.g. CD4-FITC).
- [x] The axis scales are clearly visible. Include numbers along axes only for bottom left plot of group (a 'group' is an analysis of identical markers).
- [x] All plots are contour plots with outliers or pseudocolor plots.
- [x] A numerical value for number of cells or percentage (with statistics) is provided.

Methodology

Sample preparation

EVs from plasma samples of healthy donors, P. vivax patients as well as from Fasciola
hepatica-infected cattle were isolated by size-exclusion
chromatography (SEC). EVs were coupled with Aldehyde/Sulfate Latex Beads, 4% w/v, 4 µm (Thermo Fisher), followed by incubation with antibodies. Human spleen fibroblasts (hSFs) obtained from longterm splenocytes culture from deceased transplantion donors were detached from cell culture flask, followed by incubation with antibodies.

Instrument

FacsVerse cytometer (BD) was used for beads-based FACS analysis to characterize EVs.

LSR-Fortessa cytometer (BD) was used for FACS analysis to characterize hSFs and its ICAM-1 expression.

Software

FlowJo software v10 for the analysis of FSC files derived from flow cytometry experiments performed over EV-coupled beads and hSFs.

Cell population abundance

From the homogeneity of cell morphology (FSC/SSC) and positivity for cell specific marker (CD90), cells were considered as abundant of objective cells (hSFs)

Gating strategy

For EVs characterization, first select the beads population (FSC middle and SSC high) and then based on negative control (EVs incubated with IgG isotype and 2AB), define the positive EVs population.
For hSFs characterization and ICAM-1 expression, first select the cell population, exclude debris (FSC low and SSC low), select the singlet, and then, using negative control (unstained cells), define the positive cell population.

- [x] Tick this box to confirm that a figure exemplifying the gating strategy is provided in the Supplementary Information.
