# Supplementary material for: Plasma-derived extracellular vesicles from Plasmodium vivax patients signal spleen fibroblasts via NF-kB facilitating parasite cytoadherence
Source: Nat Commun. 2020 Jun 2;11:2761. doi: 10.1038/s41467-020-16337-y (PMC7265481; doi:10.1038/s41467-020-16337-y)
Supplement: Supplementary file 4 — Description of Additional Supplementary Files [file 41467_2020_16337_MOESM4_ESM.docx]

**Description of Additional Supplementary Files**

File Name: Supplementary Data 1

Description: Clinical data of ten *P. vivax* patients at FMT-HVD, in the Amazon State, Brazil and of three *P. vivax* patients at the E.S.E Hospital San José de Tierralta, Colombia.

File Name: Supplementary Data 2

Description: Human proteins identified in circulating EVs by LC-MS/MS. Table 1. Humans proteins identified in P. vivax patients and healthy donors. Keratins, immunoglobulins and abundant plasma proteins as published in (de Menezes-Neto et al., 2015) have been removed. Table 2. Human proteins exclusively present in P. vivax patients. Table 3. Human proteins exclusively present in healthy donors. Table 4. Human proteins with differences in abundance between P. vivax patients and healthy donors. Protein abundance was estimated based on the log-transformed sum of three most abundant unique peptides per protein. A two-sided t-test was performed to assess significant changes in protein abundance. The calculated p-values were adjusted by multiple testing using the Benjamini-Hochberg method. Only proteins statistically significant are shown. Table 5. Proteins removed from the total list considered as contaminants. Categories include Bos taurus contaminants, immunoglobulins, keratins and abundant plasma proteins as reported in (de Menezes-Neto et al., 2015).

File Name: Supplementary Data 3

Description: P. vivax proteins identified in circulating EVs by LC-MS/MS. Table 1. P. vivax proteins identified in EVs from P. vivax infected patients. Descriptions of Uniprot were used to retrieve protein sequences of each entry. Blast analysis was performed in the PlasmoDB database to retrieve the corresponding gene name and description of orthologues in P. vivax SalI and PVP01 strains. Table 2. P. vivax proteins identified as false positives. Exclusion criteria was based on the detection of peptides in EVs from healthy donors.

File Name: Supplementary Data 4

Description: Gene ontology enrichment analysis of human proteins exclusively identified in PvEVs. Table 1. GO analysis for the category “Biological Process”. Table 2. GO analysis for the category “Cellular component”. Table 3. GO analysis for the category “Molecular function”. In all tables, GO terms were considered significantly enriched (highlighted) when Modified Fisher Exact P-Value was <0.05. Multiple testing corrections (Bonferroni, Benjamini and FDR) are also shown.
